# Gonadal function in patients with 47,XYY syndrome: a systematic review and meta-analysis

**DOI:** 10.1530/EC-24-0697

**Published:** 2025-03-04

**Authors:** Rossella Cannarella, Andrea Pedano, Michele Compagnone, Sandro La Vignera, Rosita A Condorelli, Aldo E Calogero

**Affiliations:** ^1^ Department of Clinical and Experimental Medicine, University of Catania, Catania, Italy; ^2^ Glickman Urological & Kidney Institute, Cleveland Clinic Foundation, Cleveland, Ohio, USA

**Keywords:** 47,XXY, Jacobs’ syndrome, testosterone, hypogonadism

## Abstract

The 47,XYY syndrome, or Jacobs syndrome, is a chromosomal disorder affecting approximately one in 1000 male births. While often asymptomatic or mildly expressed, it is associated with various physical, cognitive and behavioral features. Early studies erroneously linked the condition to aggressive behavior and elevated testosterone levels, largely based on incarcerated populations. Recent evidence contradicts this, showing testosterone levels in 47,XYY individuals are typically normal or lower than in 46,XY males. This systematic review and meta-analysis of 362 patients examine hormonal, testicular and fertility outcomes in 47,XYY syndrome. Findings reveal significantly lower testosterone levels and elevated luteinizing hormone and follicle-stimulating hormone, indicating impaired gonadal function. While testicular volumes are often normal, many patients exhibit reduced size and a notable proportion experience oligozoospermia or azoospermia. These outcomes highlight the need for counseling regarding infertility and hormonal imbalances. This review dispels the myth of 47,XYY as a ‘super-male syndrome’, emphasizing the complexity of hormonal, testicular and psychological factors. It underscores the importance of early diagnosis and a multidisciplinary approach to address endocrine and reproductive health. Regular monitoring for hypogonadism and consideration of assisted reproductive technologies are recommended to support affected individuals.

## Introduction

47,XYY syndrome, also known as Jacobs syndrome, is a genetic condition characterized by the presence of an extra Y chromosome in males, occurring in approximately one in 1000 male births (1). The condition usually arises as a random event during spermatogenesis and is not inherited. Specifically, a mistake in chromosome segregation in anaphase II of meiosis II can lead to spermatozoa with a 24,YY karyotype. If one of these atypical spermatozoa fertilizes a normal 23,X oocyte, the resulting male offspring will have a 47,XYY karyotype in each of his cells. In some instances, the extra Y chromosome may arise from nondisjunction during mitosis in the early stages of embryo development, leading to 46,XY/47,XYY mosaics (2).

The clinical features of 47,XYY syndrome are often subtle and may include tall stature, genitourinary malformations and, in some cases, behavioral or cognitive difficulties (3, 4, 5, 6, 7, 8, 9, 10). The primary focus of this review is gonadal function in 47,XYY patients, as this area remains understudied.

Epidemiological data indicate that men with 47,XYY syndrome may experience gonadal dysfunction, including small testes, impaired spermatogenesis, subfertility and infertility (11). These individuals have an increased prevalence of sperm mosaicism and aneuploidy and a higher risk of transmitting the extra Y chromosome to offspring (4). Although testicular histology and pubertal development are often normal, more recent studies suggest that fertility issues, including abnormal sperm parameters and higher rates of chromosomally abnormal spermatozoa, are relatively common (12, 13, 14, 15). Hormonal markers, such as lower serum inhibin B and higher anti-Müllerian hormone (AMH) levels, further highlight gonadal dysfunction in this population (16).

Given the emerging evidence of gonadal dysfunction in 47,XYY syndrome and the lack of consensus on its precise impact, we aim to provide a comprehensive systematic review to evaluate the available data on testicular function, focusing on serum levels of luteinizing hormone (LH), follicle-stimulating hormone (FSH), testosterone, testicular volume, sperm parameters and pregnancy rates.

## Material and methods

### Search strategy

In conducting our meta-analysis, we adhered strictly to the MOOSE guidelines for Meta-Analyses and Systematic Reviews of Observational Studies. We performed a comprehensive search on the Scopus and PubMed databases up to November 20th, 2023. The search strategy employed a combination of MeSH terms and keywords, including ‘XYY’, ‘47,XYY syndrome’, ‘XYY karyotype’, ‘Jacobs’, ‘Jacobs’ syndrome’, ‘sex chromosome abnormalities’, ‘karyotype abnormalities’, ‘chromosomes, human, Y’, ‘infertility’, ‘infertile men’, ‘testosterone blood level’, ‘LH’, ‘FSH’, ‘testis function’, ‘testis size’, ‘sperm concentration’ and ‘ART procedure’. In addition, manual searches were conducted by reviewing the reference lists of relevant studies. The search was limited to human studies, with no restrictions on language or study type.

### Selection criteria

Studies were initially assessed for inclusion based on their abstracts. If the abstract did not provide sufficient information regarding the relevance of the study to our systematic review and meta-analysis, the full text was carefully read. We included all observational, retrospective, prospective, cohort, cross-sectional, case series and case report studies. Exclusions were made for animal and *in vitro* studies, as well as reviews and meta-analyses, book chapters, editorials, letters to the editor, short surveys and studies that did not allow the extraction of the outcomes of interest. In addition, studies involving patients with mosaicism or children were excluded. Only original articles containing information on gonadal function in patients with 47,XYY karyotype were included (Table 1).

**Table 1:** Inclusion and exclusion criteria.

	Inclusion criteria	Exclusion criteria
Population	Male patients with 47,XYY karyotype post pubertal patients	Patients with mosaicism children
Comparison	Male subjects with 46,XY karyotype	
Outcome	LH, FSH, testosterone levels, testicular volumes and sperm parameters	/
Study type	Observational studies, retrospective studies, prospective studies, cross-sectional studies, case series and case reports	Animal studies, *in vitro* studies, review & meta-analyses, book chapters, editorials, letter to the editor and short survey

Abbreviations: FSH, follicle-stimulating hormone; LH, luteinizing hormone.

### Data extraction

The following data were extracted: author first name, publication year, study design, number of patients and controls, age, body mass index (BMI), LH, FSH and total testosterone (TT) values, hormone evaluation assays, testicular volumes and their evaluation methods, sperm concentration, total sperm count and data on assisted reproductive technique (ART) outcomes. Data extraction were performed by one researcher (A P) and subsequently verified by a second researcher (R C). Any discrepancy were resolved by a third, senior researcher (A E C).

### Statistical analysis

Statistical analysis was conducted using RevMan v. 5.4 (Cochrane Collaboration, UK) and Comprehensive Meta-Analysis software (version 3) (Biostat Inc., USA) for the meta-analysis of quantitative data. Statistical significance was accepted for *P*-values less than 0.05.

Serum TT levels were converted to ng/dL when reported using different units of measure. The formula provided by Wan and colleagues was used to estimate the mean and standard deviation (SD) when data were presented as the median and interquartile range (17).

To compare 47,XYY patients and controls, the mean difference (MD) was calculated. The heterogeneity index (*I*^2^) was used to assess statistical heterogeneity. Specifically, when *I*^2^ was ≤50%, the studies were considered homogeneous, and the fixed-effect model was applied to compute the pooled effect size. Conversely, when *I*^2^ was >50%, significant heterogeneity was assumed, and the random-effects model was used. Publication bias was examined qualitatively by assessing the skewness of the funnel plot, which suggested some missing studies on one side of the plot. For a quantitative analysis of publication bias, the Egger intercept test was used to determine the statistical significance of publication bias. In addition, a sensitivity analysis was conducted to test whether the results were influenced by the exclusion of individual studies.

The weighted average and 95% confidence interval were computed for each variable across all 47,XYY patients included in the study, irrespective of the presence of a control group. The prevalence of the following conditions was determined using individual patient data (mean values from the cohort were excluded): TT levels <351.3 ng/dL (18), LH levels >9.4 IU/L (18), FSH levels >12 IU/L, testicular volume <12 mL, azoospermia, sperm concentration <5 mil/mL, sperm concentration between 5 and 15 mil/mL, and total sperm count <39 mil/ejaculate.

## Results

### General results

**Figure 1:**
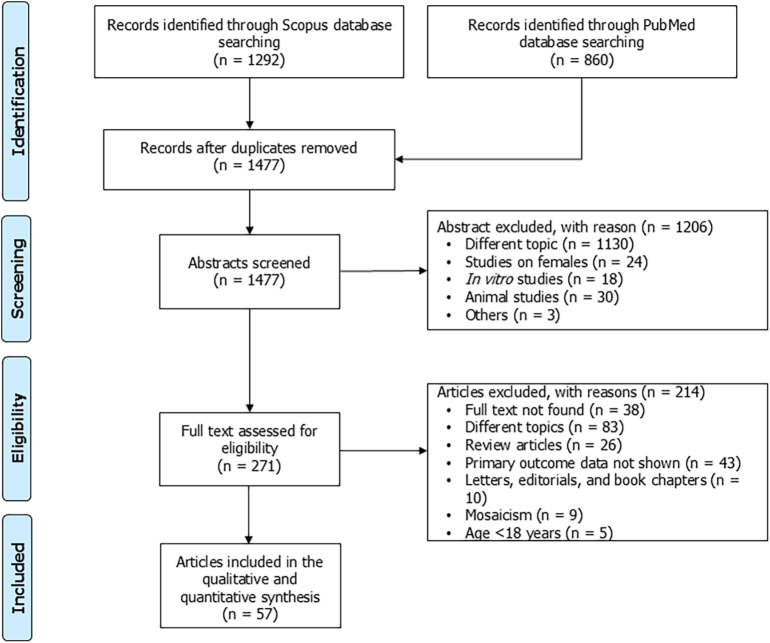
PRISMA flow chart.

Using the search strategy outlined above, 2152 articles were retrieved. After excluding 675 duplicate records, 1477 articles underwent screening. Of these, 1206 were judged not pertinent after reading the abstracts. Of the remaining 271 articles, their full text was carefully read and 214 articles were excluded. Finally, 57 studies were included in the analysis (Fig. 1).

The characteristics of the studies that met the inclusion criteria are reported in Table 2. Among the 57 included studies, 19 were controlled, involving a group of healthy men (16, 19, 20, 21, 22, 23, 24, 25, 26, 27, 28, 29, 30, 31, 32, 33, 34, 35, 36), while 38 were uncontrolled (4, 12, 15, 37, 38, 39, 40, 41, 42, 43, 44, 45, 46, 47, 48, 49, 50, 51, 52, 53, 54, 55, 56, 57, 58, 59, 60, 61, 62, 63, 64, 65, 66, 67, 68, 69, 70, 71). In terms of study design, 23 were case reports (20, 24, 25, 26, 37, 38, 40, 41, 47, 48, 49, 50, 51, 53, 54, 55, 56, 58, 60, 61, 63, 65, 66), 23 were case series (4, 12, 15, 21, 22, 23, 28, 32, 35, 42, 43, 44, 46, 52, 57, 59, 62, 64, 67, 68, 69, 70, 71, 72), eight were case-control studies (16, 28, 29, 30, 31, 33, 34, 36) and three were cohort studies (19, 39, 45). Regarding language, 47 were in English (4, 12, 15, 16, 19, 20, 21, 22, 23, 24, 25, 26, 28, 29, 30, 31, 32, 33, 34, 35, 36, 37, 38, 39, 41, 42, 43, 44, 45, 47, 49, 50, 52, 53, 54, 56, 57, 60, 63, 64, 65, 66, 67, 68, 69, 71, 72), four in Japanese (55, 58, 61, 70), three in French (40, 46, 59), two in Spanish (48, 51) and one in German (62).

**Table 2:** Main characteristics of the studies included in this meta-analysis.

First author	Year	Study design	Cases			Controls			Outcome(s)
			*n*	Age (years)	BMI (kg/m^2^)	*n*	Age (years)	BMI (kg/m^2^)	
Abdel-Razic *et al.* (44)	2012	Case series	9	30.1 ± 5.8					LH, FSH, testosterone, testicular volumes, sperm concentration, TSC
Abraham *et al.* (60)	1985	Case report	1	48					Testosterone, testicular volumes
Alonso *et al.* (51)	2005	Case report	1	45	34				LH, FSH
Baghdassarian *et al.* (29)	1975	Case control	9	21.3 ± 5.8	24.0 ± 3.7				LH, FSH, testosterone, sperm concentration, TSC
Balodimos *et al.* (71)	1966	Case series	2	66.5 ± 3.5	26.8 ± 0.4				Testicular volume
Beg *et al.* (39)	2019	Cohort study	2	41.0 ± 11.3					LH, FSH, testosterone, sperm concentration
Bennet *et al.* (59)	1987	Case series	3	30.3 ± 4.2					LH, testosterone
Blanco *et al.* (54)	2001	Case report	1	34					FSH
Blanco *et al.* (24)	1997	Case report	1						Sperm concentration
Boroujeni *et al.* (12)	2019	Case series	24	38.0 ± 6.3					LH, FSH, testosterone
Bunyan *et al.* (37)	2022	Case report	1	34					LH, FSH, testosterone, sperm concentration
Cavkaytar *et al.* (45)	2012	Cohort study	1	35					FSH
Chandley *et al.* (64)	1976	Case series	2	32.5 ± 2.1	25.5 ± 1.3				Sperm concentration
Chandley *et al.* (72)	1976	Case series	1						Sperm concentration
Chellat *et al.* (42)	2015	Case series	4						LH, FSH, testosterone, TSC
Chevret *et al.* (23)	1997	Case series	3	33.2 ± 2.6		5	29.4 ± 7.4		Sperm concentration
Davis *et al.* (16)	2020	Case control	51	13.6 ± 2.4		35	14.3 ± 2.6		LH, FSH, testosterone
El-Dahtory *et al.* (43)	2009	Case series	4	32.8 ± 4.9	20.1 ± 0.3 (2/4)				LH, FSH, testosterone, testicular volumes, sperm concentration, TSC
Faed *et al.* (63)	1982	Case report	1	28					Sperm concentration
Faed *et al.* (65)	1976	Case report	1	28	24				Sperm concentration
Han *et al.* (25)	1994	Case report	1	42					Sperm concentration, TSC
Hazama *et al.* (58)	1985	Case report	1	36	25.8				LH, FSH, testosterone, testicular volumes, sperm concentration, TSC
Ishida *et al.* (28)	1979	Case control	11	20.7 ± 10.2					LH, FSH, testosterone
Josè del Rio *et al.* (48)	2007	Case report	1	35	27				Sperm concentration, TSC
Kim *et al.* (4)	2013	Case series	3	33.0 ± 5.3	41.3 ± 6.3				LH, FSH, testosterone, testicular volumes, sperm concentration
Kjessler *et al.* (66)	1978	Case report	1	34					Testosterone, sperm concentration, TSC
Kondoh *et al.* (70)	1992	Case series	1	35					FSH, testosterone
Martin *et al.* (20)	1999	Case report	1	38					Sperm concentration
Martini *et al.* (22)	1996	Case series	2	31.0 ± 2.8					Sperm concentration
Mbamognoua *et al.* (40)	2020	Case report	1	17					LH, FSH, testosterone
Milazzo *et al.* (52)	2006	Case series	2	32 ± 2					Sperm concentration, TSC
Moretti *et al.* (47)	2007	Case report	1	30					Sperm concentration
Murakami *et al.* (55)	1997	Case report	1	32	26.8				LH, FSH, testosterone, testicular volumes, sperm concentration
Pelzmann *et al.* (30)	1976	Case control	6	23.0 ± 6.3	27.2 ± 2.5	6	22.5 ± 6.5	25.4 ± 3.1	Testosterone
Pitcher *et al.* (67)	1974	Case series	4	20.0 ± 7.3					LH
Price *et al.* (36)	1970	Case control	5	31.4 ± 3.1	-	6	30.3 ± 3.0	-	Testosterone
			12	38.8 ± 13.4	-	12	37.3 ± 15.8	-	
Ra *et al.* (41)	2020	Case report	1	32	23.2				LH, FSH, testosterone, testicular volumes, sperm concentration
Rives *et al.* (50)	2005	Case report	1	35					Testicular volumes, sperm concentration
Rives *et al.* (21)	2003	Case series	3	33.7 ± 3.8		9	33.0 ± 3.9		Sperm concentration, TSC
Scheiber *et al.* (62)	1982	Case series	1						LH, FSH, testosterone
Schiavi *et al.* (31)	1978	Case control	1	12					LH, FSH, testosterone, testicular volumes
Sciurano *et al.* (38)	2019	Case report	1	35	27.7				FSH, testosterone, testicular volumes, sperm concentration
Sharma *et al.* (33)	1975	Case control	7	23.7 ± 14.2	25.0 ± 6.8	4	32.0 ± 15.8	26.5 ± 4.8	Testosterone
Shi *et al.* (53)	2000	Case report	1	28					Sperm concentration, TSC
Siffroi *et al.* (46)	2004	Case series	5						Sperm concentration
Skakkebaek *et al.* (32)	1973	Case series	8	28.4 ± 7.2					Testicular volumes, sperm concentration, TSC
Skakkebaek *et al.* (35)	1973	Case series	4	21.0					Sperm concentration
Skakkebaek *et al.* (69)	1970	Case series	2	30.5 ± 9.2	25.9 ± 1.4				Sperm concentration
Solari *et al.* (56)	1997	Case report	1	29					Testicular volumes, sperm concentration
Speed *et al.* (26)	1991	Case report	1						FSH, sperm concentration
Terada *et al.* (61)	1984	Case report	1	29	21.7				LH, FSH, testosterone
Wakeling *et al.* (34)	1973	Case control	11	25.6 ± 8	23.2 ± 3.9	6	30.3 ± 7.6	23.8 ± 2.4	LH, testosterone
Wong *et al.* (49)	2007	Case report	1	29					Sperm concentration
Yoshida *et al.* (57)	1997	Case series	3	34.3 ± 2.1					LH, FSH, testosterone, testicular volumes, sperm concentration, TSC
Zeuthen *et al.* (68)	1973	Case series	5		22.6 ± 2.8				Testosterone, testicular volumes
Zhang *et al.* (15)	2020	Case series	21	28.0 ± 5.2					LH, FSH, testosterone, sperm concentration
Zhao *et al.* (19)	2022	Cohort study	143		30.2 ± 5.7	*		27.9 ± 4.2	Testosterone

Abbreviations: BMI, body mass index; FSH, follicle-stimulating hormone; LH, luteinizing hormone; TSC, total sperm count.

* Unclear information.

Serum testosterone levels were measured using electrochemiluminescence (ECL) in one study (15), chemiluminescence (CL) in two studies (12, 44), liquid chromatography–tandem mass spectrometry in one study (16), chromatography in two studies (36, 68), competitive protein binding analysis in three studies (29, 31, 34), and radioimmunoassay (RIA) in seven studies (28, 30, 33, 57, 59, 62, 66). No description of the method was provided in 14 studies (4, 19, 37, 38, 39, 40, 41, 42, 43, 45, 55, 58, 60, 61). Serum LH and FSH levels were assessed by ECL in one study (15), CL in two studies (12, 44), and RIA in nine studies (28, 29, 31, 34, 57, 59, 62, 66, 67). The levels of these hormones were not reported in 18 studies (4, 16, 26, 37, 38, 39, 40, 41, 42, 43, 45, 51, 54, 55, 58, 60, 61, 70). Finally, testicular volumes were measured using scrotal ultrasound in four studies (4, 41, 44, 50) and an orchidometer in four studies (35, 57, 66, 68), with no information provided in five studies (38, 55, 56, 58, 60).

### Analysis of confounders: age and body mass index

Age did not differ significantly between patients with 47,XYY syndrome and controls (MD −0.57 years, 95% CI: −1.59, 0.44; *P* = 0.27). No inter-study heterogeneity was observed (*I*^2^ = 0%; *P* = 0.74) (Supplementary Fig. 1A (see section on Supplementary materials given at the end of the article)). In the sensitivity analysis, no study was found sensitive enough to change the conclusion that age does not differ significantly between patients and controls (Supplementary Fig. 2A). The analysis revealed no publication bias, as indicated by Egger’s test (intercept 0.55, 95% CI: −0.71, 1.81; *P* = 0.32) and the symmetry of the funnel plot (Supplementary Fig. 2B). Finally, the weighted mean age of the 192 patients with 47,XYY syndrome was 20.9 years (95% CI 20.4; 21.4) (Supplementary Fig. 3A).

BMI did not differ significantly between patients with 47,XYY syndrome and controls (MD 0.30 kg/m^2^, 95% CI: −1.78, 2.38, *P* = 0.78). No inter-study heterogeneity was observed (*I*^2^ = 0%; *P* = 0.50) (Supplementary Fig. 1B). In the sensitivity analysis, no individual study was found to be sensitive enough to change the conclusion that BMI was not significantly different between patients and controls (Supplementary Fig. 2C). The analysis revealed no publication bias, as indicated by the symmetry of the funnel plot and Egger’s test (intercept 1.77, 95% CI: −89.0, 92.6; *P* = 0.84) (Supplementary Fig. 2D). Furthermore, the weighted mean BMI of the 201 patients with 47,XYY syndrome was 27.0 kg/m^2^ (95% CI 26.6; 27.4) (Supplementary Fig. 3B).

### Study outcomes

#### Case-control analysis (meta-analysis)

**Figure 2:**
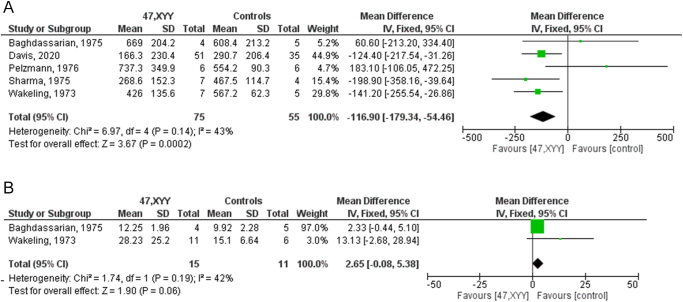
Serum levels of TT (panel A) and LH (panel B) in patients with 47,XYY karyotype compared to 46,XY controls. Data from the following studies were included: Baghdassarian *et al.* (29); Davis *et al.* (16); Pelzmann *et al.* (30); Sharma *et al.* (33); Wakeling *et al.* (34).

Serum TT levels were significantly lower in 47 patients with XYY syndrome compared to healthy controls (MD −116.90 ng/dL, 95% CI −179.34; −54.46; *P* = 0.0002), with no inter-study heterogeneity observed (*I*^2^ = 43%; *P* = 0.14) (Fig. 2A). In the sensitivity analysis, one study was found to be sensitive enough to change the results (16) (SMD −0.35, CI −0.99, 0.28; *P* = 0.28) (Supplementary Fig. 4A). No publication bias was detected, as indicated by the symmetry of the funnel plot and Egger’s test (intercept 0.32, 95% CI: −4.67, 5.31; *P* = 0.85) (Supplementary Fig. 4B).

An upward trend in serum LH levels was observed in 47,XYY patients compared to 46,XY controls (MD 2.65 IU/L, 95% CI −0.08; 5.38; *P* = 0.06), with no inter-study heterogeneity (*I*^2^ = 42%; *P* = 0.19) (Fig. 2B). In the sensitivity analysis, no individual study was found sensitive enough to change the conclusion that LH is not significantly different between patients and controls (Supplementary Fig. 4C). No publication bias was detected, as indicated by the symmetry of the funnel plot. A quantitative analysis of publication bias using Egger’s test could not be performed, as only two studies were included (Supplementary Fig. 4D).

Regarding the other outcomes, particularly FSH, testicular volume, sperm concentration and total sperm count, the controlled studies that included these data were insufficient to perform a meta-analysis.

#### Analysis of disease prevalence

**Figure 3:**
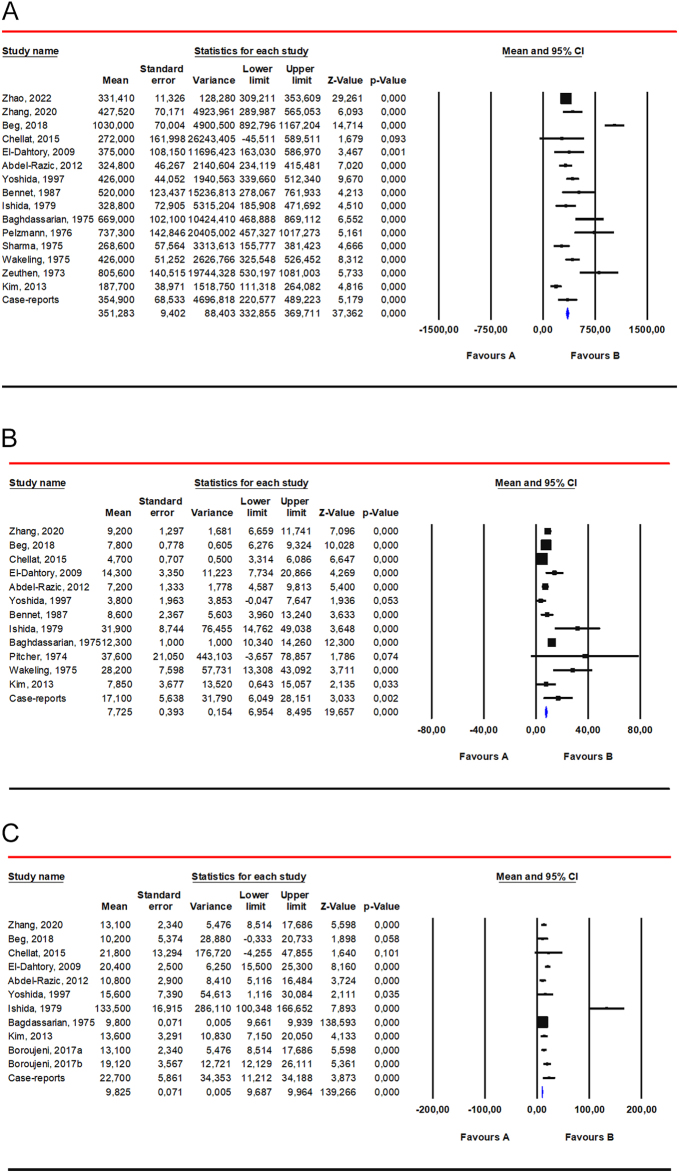
Weighted mean of serum hormone levels. (panel A) Weighted mean of TT. Data from the following studies were included: Zhao *et al.* (19); Zhang *et al.* (15); Beg *et al.* (39); Chellat *et al.* (42); El-Dahtory & Elsheikha (43); Abdel-Razic *et al.* (44); Yoshida *et al.* (57); Bennet *et al.* (59); Ishida *et al.* (28); Baghdassarian *et al.* (29); Pelzmann *et al.* (30); Sharma *et al.* (33); Wakeling *et al.* (34); Zeuthen *et al.* (68); Kim *et al.* (4). (panel B) Weighted mean of LH. Data from the following studies were included: Zhang *et al.* (15); Beg *et al.* (39); Chellat *et al.* (42); El-Dahtory & Elsheikha (43); Abdel-Razic *et al.* (44); Yoshida *et al.* (57); Bennet *et al.* (59); Ishida *et al.* (28); Baghdassarian *et al.* (29); Pitcher *et al.* (67); Wakeling *et al.* (34); Kim *et al.* (4). (panel C) Weighted mean of FSH. Data from the following studies were included: Zhang *et al.* (15); Beg *et al.* (39); Chellat *et al.* (42); El-Dahtory & Elsheikha (43); Abdel-Razic *et al.* (44); Yoshida *et al.* (57); Ishida *et al.* (28); Baghdassarian *et al.* (29); Kim *et al.* (4); Boroujeni *et al.* (12).

The weighted mean serum TT level was 351.3 ng/dL (95% CI 332.9; 369.7) based on data from 228 patients with 47,XYY syndrome (Fig. 3A). When considering only case reports and case series (where individual values were available), TT levels were less than 345.8 ng/dL in 38.9% of cases (35 out of 90).

The weighted mean serum LH level was 7.7 IU/L (95% CI 7.0; 8.5) based on data from 76 patients with 47,XYY karyotype (Fig. 3B). In case reports and case series (where individual values were available), LH levels were greater than 9.4 IU/L in 53.9% of cases (41 out of 76).

The weighted mean serum FSH level was 9.8 IU/L (95% CI 9.7; 10.0) based on data from 61 patients with 47,XYY karyotype (Fig. 3C). When considering only case reports and case series (where individual values were available), FSH levels were greater than 12 IU/L in 56.5% of cases (35 out of 62).

**Figure 4:**
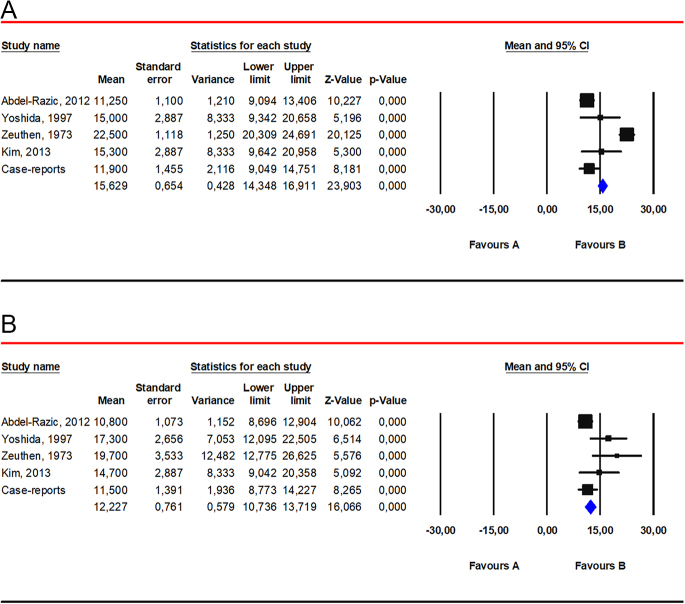
Weighted mean testicular volume. (panel A) Weighted mean right testicular volume. Data from the following studies were included: Abdel-Razic *et al.* (44); Yoshida *et al.* (57); Zeuthen *et al.* (79); Kim *et al.* (4). (panel B) Weighted mean left testicular volume. Data from the following studies were included: Abdel-Razic *et al.* (44); Yoshida *et al.* (57); Zeuthen *et al.* (79); Kim *et al.* (4).

The weighted mean right testicular volume was 15.6 mL (95% CI 14.3; 16.9) based on data from 25 patients with 47,XYY karyotype (Fig. 4A). When considering only case reports and case series (where individual values were available), right testicular volume was less than 12 mL in 28% of cases (seven out of 25).

The weighted mean left testicular volume was 12.2 mL (95% CI 10.7; 13.7) based on data from 26 patients with 47,XYY karyotype (Fig. 4B). In case reports and case series (where individual values were available), left testicular volume was less than 12 mL in 38.5% of cases (ten out of 26).

**Figure 5:**
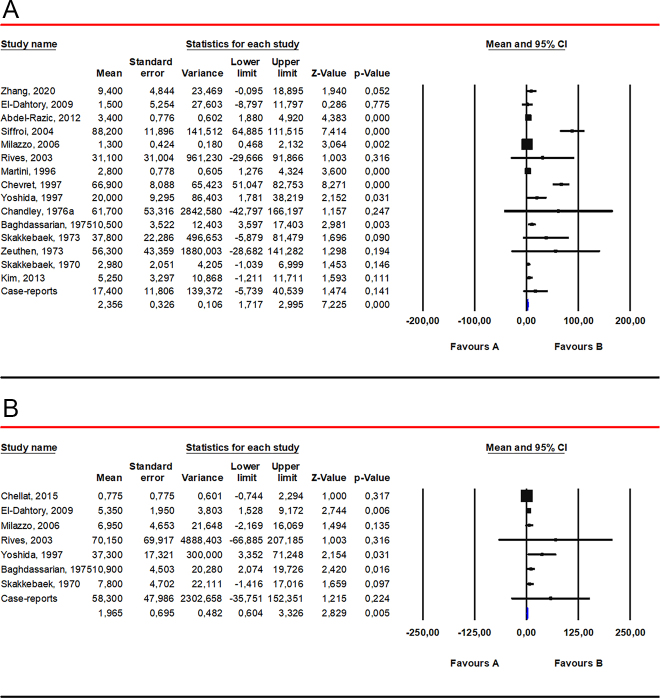
Weighted mean sperm concentration and total sperm count. (panel A) Weighted mean sperm concentration. Data from the following studies were included: Zhang *et al.* (15); El-Dahtory & Elsheikha (43); Abdel-Razic *et al.* (44); Siffroi *et al.* (46); Milazzo *et al.* (52); Rives *et al.* (21); Martini *et al.* (22); Chevret *et al.* (23); Yoshida *et al.* (57); Chandley *et al.* (64); Baghdassarian *et al.* (29); Skakkebaek *et al.* (32); Zeuthen *et al.* (79); Skakkebaek *et al.* (69); Kim *et al.* (4). (panel B) Weighted mean total sperm count. Data from the following studies were included: Chellat *et al.* (42); El-Dahtory & Elsheikha (43); Milazzo *et al.* (52); Rives *et al.* (21); Yoshida *et al.* (57); Baghdassarian *et al.* (29); Skakkebaek *et al.* (69).

The weighted mean sperm concentration was 2.4 mil/mL (95% CI 1.7; 3.0) based on data from 89 patients with 47,XYY karyotype (Fig. 5A). When considering only case reports and case series (where individual values were available), 8.1% were azoospermic (7/86), 45.3% had severe oligozoospermia (<5 mil/mL; 39/86) and 16.3% had a sperm concentration between 5 and 15 mil/mL (14/86).

The weighted mean total sperm count was 2.0 mil/ejaculate (95% CI 0.6; 3.3) based on data from 28 patients with 47,XYY karyotype (Fig. 5B). Considering only case reports and case series (where individual values were available), 23.3% were azoospermic (7/30) and 66.7% had oligozoospermia (<39 mil/ejaculate: 20/30).

## Discussion

The analysis presented in this article demonstrates that patients with 47,XYY syndrome exhibit lower serum TT levels (mean: 116.90 ng/dL) compared to controls. Importantly, no significant differences in age or BMI were observed between the two groups, suggesting that the lower TT levels in 47,XYY patients cannot be attributed to these factors. This finding highlights that the reduced TT levels in this population are likely independent of age and BMI, suggesting other potential underlying mechanisms contribute to hormonal alterations in 47,XYY syndrome. The weighted mean serum TT level is 351.3 ng/dL. These findings challenge the longstanding assumption that testosterone levels are elevated in patients with 47,XYY karyotype, suggesting instead that their TT levels are either normal or reduced.

The low testosterone levels observed in men with 47,XYY syndrome may be attributed to several molecular mechanisms. The extra Y chromosome leads to an overexpression of genes located on the Y chromosome, including the *SRY* gene, which could disrupt normal testicular function and testosterone production. In addition, extra sex chromosomes can cause chromosome instability and dysregulation of gene expression (73, 74). As such, the presence of an extra Y chromosome may impact the expression of genes involved in steroidogenesis, potentially disrupting the enzymes responsible for testosterone synthesis. The presence of an extra chromosome could also influence gene expression through epigenetic modifications, as demonstrated for other chromosomal disorders (75). In turn, epigenetic changes, such as DNA methylation or histone modifications, could influence the expression of genes regulating testosterone production. Testicular dysfunction is common in 47,XYY males (12), with impaired Leydig cell function potentially contributing to reduced testosterone synthesis. Furthermore, lower levels of inhibin B and higher levels of AMH are often observed in these patients, reflecting gonadal dysfunction that may also impact testosterone levels (16). Together, these factors suggest that the presence of an extra Y chromosome disrupts multiple aspects of testicular function, leading to lower testosterone levels in 47,XYY males. Further research is needed to fully understand these mechanisms. Historically, 47,XYY syndrome has been viewed as a ‘super male’ condition associated with robust testicular function, largely due to early studies conducted in penal institutions. In these settings, patients with 47,XYY karyotype were often linked to aggressive and antisocial behavior. These studies reported elevated testosterone levels in these patients, reinforcing the perception of heightened masculinity. The first article in which Richard H W Jacobs and his colleagues explored the characteristics associated with 47,XYY karyotype and introduced the term ‘supermale’ was published in 1965 (76). The article examined the potential link between the extra Y chromosome and behavioral traits such as aggression or criminality. The authors proposed that the additional Y chromosome might increase the likelihood of exhibiting traits traditionally associated with masculinity, including heightened aggression or impulsivity. The article also proposed that the extra Y chromosome could influence the development of physical features commonly observed in men, such as increased height and muscularity, which are partially regulated by testosterone (76).

Although Jacobs and colleagues initially proposed that patients with 47,XYY syndrome had elevated testosterone levels, subsequent evidence has contradicted this hypothesis. Over time, numerous studies have shown that the elevated testosterone levels observed in 47,XYY patients within prison or psychiatric settings were comparable to those in men with the 46,XY karyotype. Moreover, non-institutionalized patients with 47,XYY karyotype exhibited testosterone levels comparable to those of healthy controls. Specifically, Price and colleagues (36) demonstrated no significant difference in serum testosterone levels between 47,XYY patients and 46,XY men. In addition, Sharma and colleagues (33) conducted the first direct study of testosterone production rate, which revealed a decreased, rather than increased, testosterone production in patients with 47,XYY.

Subsequent studies have increasingly focused on serum testosterone levels in patients with chromosomal abnormalities, particularly examining the relationship between testosterone and aggressive behaviors. However, beyond hormone levels, research has also emphasized the significant role of factors such as education, life experiences and family dynamics in shaping the behavior of patients with 47,XYY syndrome. These findings suggest that the presence of an extra Y chromosome does not inherently predispose individuals to violent behavior. Witkin and colleagues (77) found that patients with 47,XYY had significantly lower scores on intelligence tests and lower educational attainment. Similarly, among 46,XY men, those with criminal convictions had notably lower intelligence test scores and educational levels compared to those without convictions. However, when controlling for variables such as parental socioeconomic status, the higher crime rate among patients with 47,XYY syndrome was significantly reduced. Schiavi *et al.* (27) highlighted that data from social records, psychological interviews and projective tests did not support the notion that 47,XYY patients are more violent or aggressive than the general population. In addition, elevated levels of testosterone, LH and FSH in the 47,XYY group did not appear to be specifically associated with delinquent or aggressive behavior.

The results of this meta-analysis show a trend towards higher serum LH levels. The weighted mean LH and FSH levels were 7.7 IU/L and 9.8 IU/L, respectively. When considering only case reports and case series (where individual values were provided), more than half of the cases exhibited LH levels greater than 9.4 IU/L and FSH levels exceeding 12 IU/L. However, the available data are insufficient to perform a meta-analysis on other variables, such as FSH levels, right and left testicular volume, sperm concentration and total sperm count.

Data on sperm parameters are derived from a smaller number of cases. The weighted mean sperm concentration and total sperm count were 2.4 mil/mL and 2.0 mil/ejaculate, respectively. Of the cases, 8.1% were azoospermic, 30.2% had normal sperm concentration, and the remaining patients with 47,XYY syndrome exhibited varying degrees of oligozoospermia, as defined by the sixth edition of the WHO manual for human semen analysis. These findings align with data presented in recent reviews on the topic (13, 78).

Zhang *et al.* (15) and Boroujeni *et al.* (12) evaluated the outcomes of ART in patients with 47,XYY syndrome. The authors reported three ART cases: one ICSI procedure resulting in the birth of a healthy boy, one IVF leading to the birth of a healthy boy, and one IVF with no clinical pregnancy. Boroujeni *et al.* documented a total of 11 ART procedures, which resulted in five biochemical pregnancies and at least three live births.

Some studies have also investigated urinary testosterone levels, comparing them with blood testosterone levels; however, urinary testosterone values were not included in this meta-analysis. A potential bias in the analysis could stem from the use of different methods for measuring serum testosterone, such as electrochemiluminescence, chemiluminescence, liquid chromatography–tandem mass spectrometry, chromatography, competitive protein binding analysis and radioimmunoassay. These methodological differences may introduce challenges when comparing testosterone values. Furthermore, many older studies did not specify the methods used for testosterone measurement, which could contribute to inconsistencies in the data.

The data obtained highlight the potential for improving counseling for patients with 47,XYY syndrome. Diagnosis is often made through karyotype analysis as part of the evaluation for male infertility. The altered spermatogenesis associated with 47,XYY makes natural conception more difficult, leading to the frequent recommendation of ART and sperm cryopreservation. However, the overall analysis underscores the importance of considering hypogonadism not only in terms of infertility but also with regard to hypotestosteronemia. These patients should be monitored over time, regardless of fertility concerns, with attention to clinical aspects related to serum testosterone levels. Identifying hypotestosteronemia and, when necessary, implementing hormone replacement therapy is crucial. Such therapy is beneficial not only for sexual function but also for the numerous systemic roles that testosterone plays in the body.

In conclusion, this study examines a cohort of 362 patients with 47,XYY syndrome, representing the largest group available in the literature to date. The database primarily consists of case reports and case series, with only a few case–control studies comparing 47,XYY patients to healthy controls. Furthermore, only two studies are prospective, while the remainder are retrospective or observational. Our findings indicate that serum testosterone levels in patients with 47,XYY karyotype are either normal or low, challenging the long-standing belief that these patients have elevated testosterone levels. In addition, a trend towards higher LH levels was observed. Finally, consistent with previous studies, we confirm the presence of impaired spermatogenesis in this cohort. Based on these findings, counseling should not only address infertility but also include an evaluation of potential hypogonadism onset.

## Supplementary materials



## Declaration of interest

The authors declare that there is no conflict of interest that could be perceived as prejudicing the impartiality of the work reported.

## Funding

This work did not receive any specific grant from any funding agency in the public, commercial or not-for-profit sector.

## Attestation statements

The subjects in this trial have not concomitantly been involved in other randomized trials. Data regarding any of the subjects in the study have not been previously published unless specified. Data will be made available to the editors of the journal for review or query upon request.

## Data sharing statement

Data will be shared upon request to the corresponding author.
