# Supplementary material for: Gonadal function in patients with 47,XYY syndrome: a systematic review and meta-analysis
Source: Endocr Connect. 2025 Mar 4;14(4):e240697. doi: 10.1530/EC-24-0697 (PMC11906151; doi:10.1530/EC-24-0697)
Supplement: Supplementary file 1 [file supplementary_materials.pdf]

A

Study or Subgroup	47,XYY Mean	47,XYY SD	47,XYY Total	Controls Mean	Controls SD	Controls Total	Weight	Mean Difference IV, Fixed, 95% CI
Chevret, 1997	34.5	3.5	2	29.4	7.4	5	1.6%	5.10 [-3.00, 13.20]
Davis, 2020	13.6	2.4	51	14.3	2.6	35	88.0%	-0.70 [-1.78, 0.38]
Pelzmann, 1976	23	6.4	6	22.5	6.5	6	1.9%	0.50 [-6.80, 7.80]
Price, 1970a	31.4	6.9	5	30.3	7.3	6	1.5%	1.10 [-7.31, 9.51]
Price, 1970b	38.8	13.4	12	37.3	15.8	12	0.8%	1.50 [-10.22, 13.22]
Rives, 2003	33.7	3.8	3	33	3.9	10	4.3%	0.70 [-4.23, 5.63]
Sharma, 1975	23.7	14.2	7	32	15.9	4	0.3%	-8.30 [-27.10, 10.50]
Wakeling, 1973	25.6	8	11	30.3	7.6	6	1.7%	-4.70 [-12.40, 3.00]
**Total (95% CI)**			**97**			**84**	**100.0%**	**-0.57 [-1.59, 0.44]**

Heterogeneity: Chi² = 4.30, df = 7 (P = 0.74); I² = 0%

Test for overall effect: Z = 1.11 (P = 0.27)

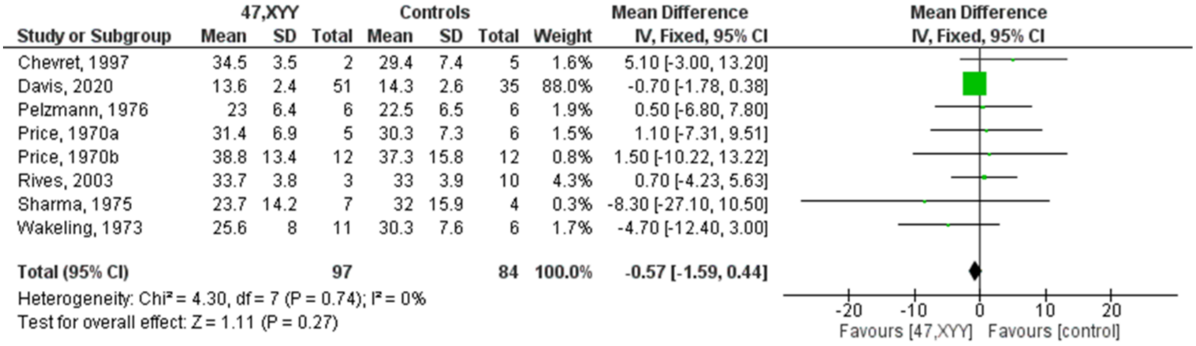

B

Study or Subgroup	47,XYY Mean	47,XYY SD	47,XYY Total	Controls Mean	Controls SD	Controls Total	Weight	Mean Difference IV, Fixed, 95% CI
Pelzmann, 1976	27.17	2.54	6	25.44	3.07	6	42.4%	1.73 [-1.46, 4.92]
Sharma, 1975	25.01	6.8	7	26.54	4.81	4	9.1%	-1.53 [-8.43, 5.37]
Wakeling, 1973	23.22	3.86	11	23.83	2.4	6	48.5%	-0.61 [-3.59, 2.37]
**Total (95% CI)**			**24**			**16**	**100.0%**	**0.30 [-1.78, 2.38]**

Heterogeneity: Chi² = 1.40, df = 2 (P = 0.50); I² = 0%

Test for overall effect: Z = 0.28 (P = 0.78)

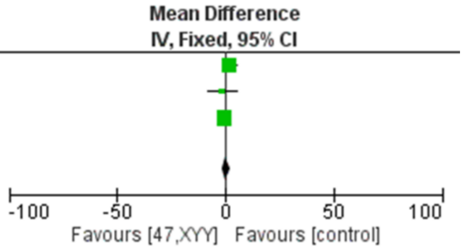

Supplementary Figure 1. Age **(Panel A)** and body mass index **(Panel B)** in patients with 47,XYY syndrome compared to 46,XY controls. Data from the following studies were included: Chevret et al., 1997 (31); Davis et al., 2020(17); Pelzmann et al., 1976 (37); Price et al., 1970 (22); Rives et al., 2003 (29); Sharma et al., 1975 (40); Wakeling et al., 1973 (41).

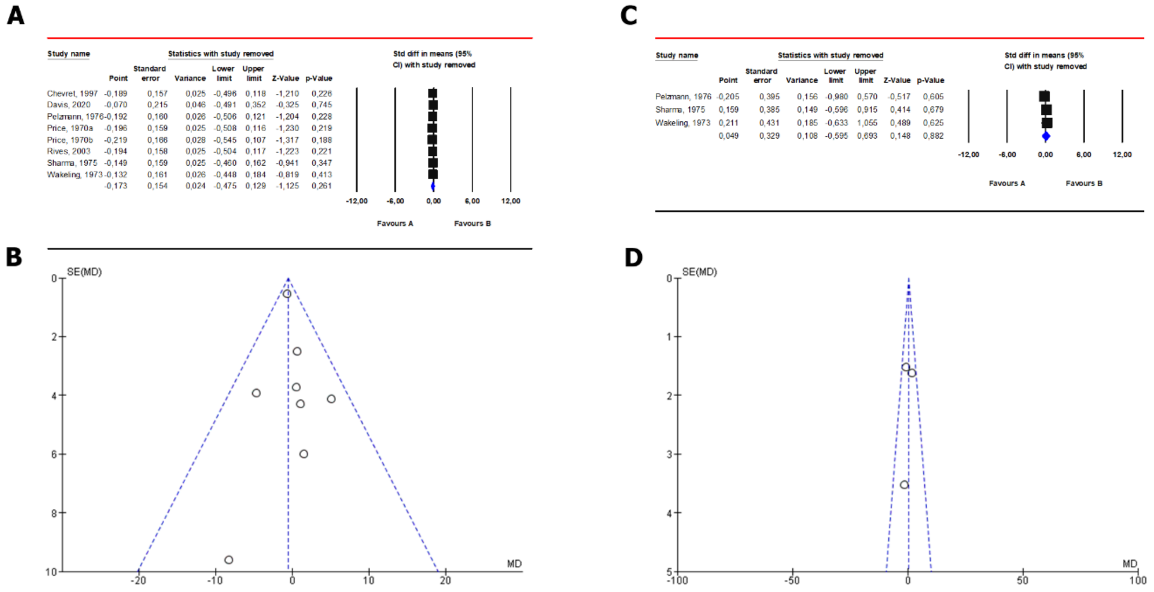

Supplementary Figure 2. Age and body mass index (BMI) in patients with 47, XYY syndrome compared to 46,XY controls. **Panel A**. Sensitivity analysis of age. **Panel B**. Publication bias of age. **Panel C**. Sensitivity analysis of BMI. **Panel D**. Publication bias of BMI. Data from the following studies were included: Chevret et al., 1997 (31); Davis et al., 2020 (17); Pelzmann et al., 1976 (37); Price et al., 1970 (22); Rives et al., 2003 (29); Sharma et al., 1975 (40); Wakeling et al., 1973 (41).

A

Study name	Mean	Standard error	Variance	Lower limit	Upper limit	Z-Value	p-Value
Zhang, 2020	28,000	1,135	1,288	25,776	30,224	24,675	0,000
Boroujeni, 2017a	38,300	1,704	2,904	34,960	41,640	22,475	0,000
Boroujeni, 2017b	37,700	2,033	4,134	33,715	41,685	18,541	0,000
Beg, 2018	41,000	7,990	63,845	25,339	56,661	5,131	0,000
Davis, 2020	13,600	0,336	0,113	12,941	14,259	40,468	0,000
El-Dahtory, 2009	30,100	1,933	3,738	26,311	33,889	15,569	0,000
Abdel-Razic, 2012	30,100	1,933	3,738	26,311	33,889	15,569	0,000
Milazzo, 2006	32,000	1,980	3,920	28,119	35,881	16,162	0,000
Rives, 2003	33,700	2,194	4,813	29,400	38,000	15,361	0,000
Martini, 1996	31,000	1,980	3,920	27,119	34,881	15,657	0,000
Chevret, 1997	34,500	2,475	6,125	29,649	39,351	13,940	0,000
Yoshida, 1997	34,300	1,212	1,470	31,924	36,676	28,290	0,000
Bennet, 1987	30,300	2,425	5,880	25,547	35,053	12,496	0,000
Ishida, 1979	20,700	3,075	9,458	14,672	26,728	6,731	0,000
Chandley, 1976a	32,500	1,485	2,205	29,590	35,410	21,887	0,000
Baghdassarian, 1975	21,300	1,933	3,738	17,511	25,089	11,017	0,000
Pelzmann, 1976	23,000	2,613	6,827	17,879	28,121	8,803	0,000
Shakkebek, 1973	28,400	2,546	6,480	23,411	33,389	11,157	0,000
Pitcher, 1974	20,000	3,650	13,323	12,846	27,154	5,479	0,000
Sharma, 1975	23,700	5,367	28,806	13,181	34,219	4,416	0,000
Wakeling, 1975	25,600	2,412	5,818	20,872	30,328	10,613	0,000
Shakkebaek, 1970	30,500	6,505	42,320	17,750	43,250	4,688	0,000
Price, 1970	38,800	3,868	14,963	31,218	46,382	10,030	0,000
Kim, 2013	33,000	3,060	9,363	27,003	38,997	10,784	0,000
Balodimos, 1966	66,500	2,475	6,125	61,649	71,351	26,870	0,000
Case-reports	31,600	1,424	2,028	28,809	34,391	22,189	0,000
	20,918	0,260	0,068	20,407	21,428	80,368	0,000

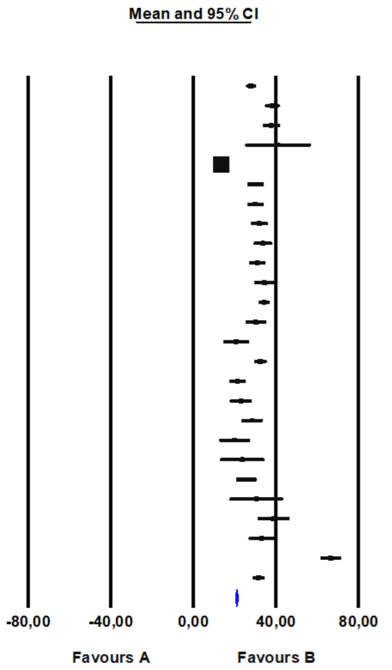

B

Study name	Mean	Standard error	Variance	Lower limit	Upper limit	Z-Value	p-Value
Zhao, 2022	30,200	0,477	0,227	29,266	31,134	63,358	0,000
Chandley, 1976a	25,500	0,919	0,845	23,698	27,302	27,740	0,000
Baghdassarian, 1975	24,000	1,233	1,521	21,583	26,417	19,459	0,000
Pelzmann, 1976	27,200	1,021	1,042	25,200	29,200	26,650	0,000
Sharma, 1975	25,000	2,570	6,606	19,963	30,037	9,727	0,000
Wakeling, 1975	23,200	1,176	1,383	20,895	25,505	19,730	0,000
Zeuthen, 1973	22,600	1,252	1,568	20,146	25,054	18,048	0,000
Shakkebaek, 1970	25,900	0,990	0,980	23,960	27,840	26,163	0,000
Kim, 2013	41,300	3,695	13,653	34,058	48,542	11,177	0,000
Balodimos, 1966	26,800	0,283	0,080	26,246	27,354	94,752	0,000
Case-reports	24,700	1,236	1,528	22,277	27,123	19,981	0,000
	27,026	0,209	0,044	26,617	27,435	129,506	0,000

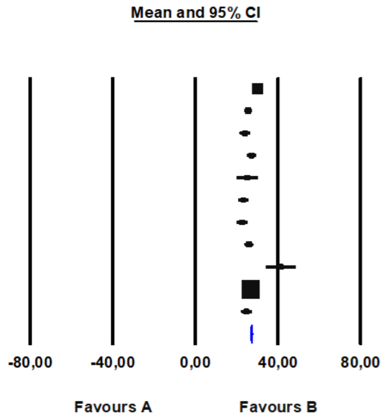

Supplementary Figure 3. Weighted mean age (**Panel A**) and body mass index (BMI) (**Panel B**). Data from the following studies were included: Zhang et al., 2020 (16); Boroujeni et al., 2017 (13); Beg et al., 2018 (45); Davis et al., 2020 (17); El-Dahtory et al., 2009 (49); Abdel-Razic et al., 2012 (50); Milazzo et al., 2006 (58); Rives et al., 2003 (29); Martini et al., 1996 (30); Chevret et al., 1997 (31); Yoshida et al., 1997 (63); Bennet et al., 1987 (65); Ishida et al., 1979 (35); Chandley et al., 1976 (70); Baghdassarian et al., 1975 (36);

Pelzmann et al., 1976 (37); Skakkebek et al., 1973 (39); Pitcher et al., 1974 (73); Sharma et al., 1975 (40); Wakeling et al., 1973 (41); Skakkebek et al., 1970 (75); Price et al., 1970 (22); Kim et al., 2013 (76); Balodimos et al., 1979 (78); Zhao et al., 2022 (23); Chandley et al., 1976 (70); Baghdassarian et al., 1975 (36); Zeuthen et al., 1973 (80).

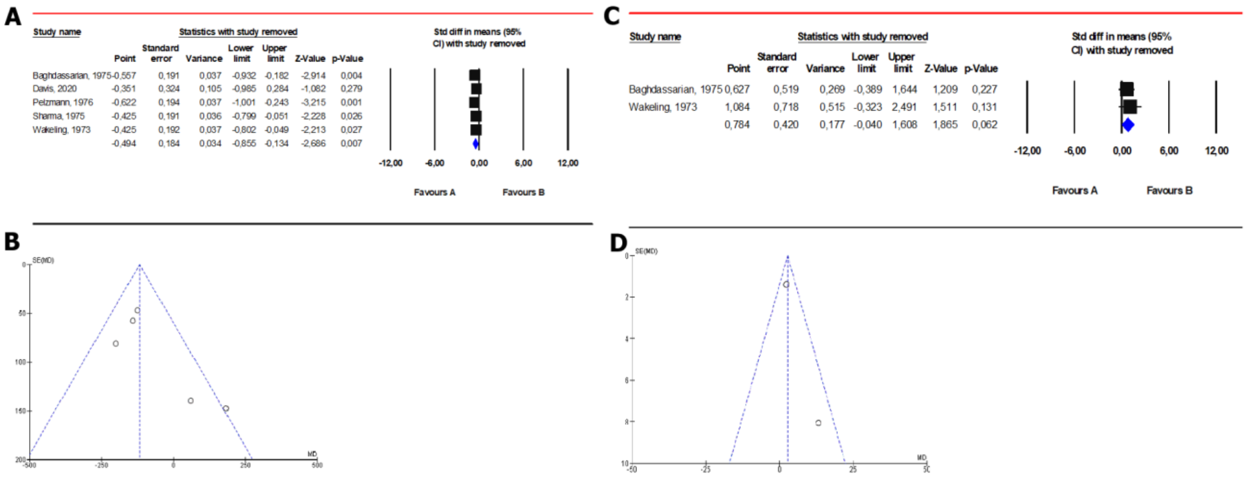

Supplementary Figure 4. Serum levels of total testosterone and luteinizing hormone (LH) in patients with 47,XYY karyotype compared to 46,XY controls. **Panel A.** Sensitive analysis of total testosterone. **Panel B**. Analysis of publication bias of total testosterone. Data from the following studies were included: Baghdassarian et al., 1975 (36); Davis et al., 2020(17); Pelzmann et al., 1976 (37); Sharma et al., 1975 (40); Wakeling et al., 1973 (41). **Panel C.** Sensitive analysis of LH. **Panel D**. LH analysis of publication bias. Data from the following studies were included: Baghdassarian et al., 1975 (36); Wakeling et al., 1973 (41).
